# A Linnaeus NG interactive key to the species of *Glomera* (Orchidaceae, Coelogyninae) from Southeast Asia

**DOI:** 10.3897/phytokeys.110.28435

**Published:** 2018-10-26

**Authors:** Richa Kusuma Wati, Rogier R. van Vugt, Barbara Gravendeel

**Affiliations:** 1 Naturalis Biodiversity Center, Endless Forms group, P.O. Box 9517, Leiden, The Netherlands; 2 Center for Plant Conservation, Bogor Botanic Gardens-LIPI, Jalan Ir. H. Juanda 13, Bogor, Indonesia; 3 Leiden University, Hortus botanicus, P.O. Box 9500, Leiden, The Netherlands; 4 Institute Biology Leiden, Leiden University, P.O. Box 9505, Leiden, The Netherlands

**Keywords:** Cybertaxonomy, epiphytes, Indonesia, monitoring, necklace orchids, Papua New Guinea, Taksonomi Siber, epifit, Indonesia, pengamatan, anggrek kalung, Papua New Guinea

## Abstract

We present a multilingual interactive key available online (http://glomera.linnaeus.naturalis.nl) that can be used on any web browser without the need for installing additional software. The key includes 169 species of *Glomera*, a genus within the necklace orchids (Coelogyninae) not yet comprehensively treated in any recent field guide or web-based survey. With this key, plants can be identified using a combination of vegetative and floristic characters in addition to distribution and ecology as a first step to further taxonomic revisions. We urge anyone with an interest in wild orchids in Southeast Asia to contribute new observations to update current information on the distribution of these overlooked plants as a first step for a taxonomic revision and to gain more insight into their conservation status.

## Abstract (id)

Studi ini menyajikan kunci identifikasi interaktif multibahasa yang dapat diakses secara online (http://glomera.linnaeus.naturalis.nl) dan dapat digunakan pada berbagai jenis peramban web tanpa perlu menggunakan aplikasi tambahan. Kunci identifikasi ini terdiri dari 169 jenis *Glomera* dalam genus anggrek kalung (Coelogyninae) yang belum pernah dibahas secara menyeluruh dalam panduan lapangan atau survei berbasis online. Tumbuhan dapat diidentifikasi menggunakan kombinasi karakter vegetatif dan bunga juga distribusi dan ekologi sebagai langkah pertama untuk revisi taksonomi lebih lanjut. Kami menghimbau bagi siapapun yang mempunyai ketertarikan dengan anggrek liar di Asia Tenggara untuk berkontribusi memberikan informasi terbaru dan menambahkan data distribusi dari spesies ini sebagai langkah awal untuk merevisi taksonomi dan untuk mendapatkan lebih banyak informasi tentang status konservasi spesies ini.

## Introduction

Southeast Asia is one of the richest biodiversity regions on earth. Its complex geological history contributed to unique biota and high concentration of endemic species (Myers et al. 2000). The region also suffered the highest rate of habitat loss and associated biodiversity due to deforestation and global warming (Carr 2004; Sodhi and Brook 2006; Sodhi et al. 2010). More than 7 million hectares of forests were lost per year between 2000 and 2010 to meet rising demands for food, fuel and fibres (FAO 2016). Global warming accelerates the current biodiversity crisis that will especially lead to the extinction of those species living on mountain tops (Spehn et al. 2010). To measure the impact of deforestation and global warming on the species level, biodiversity indicators are very useful to monitor local biodiversity losses (Caro and O’Doherty 1999; Lindenmayer et al. 1999; Soberόn et al. 2000; Kati et al. 2004). Orchids are an ideal flagship group to investigate biodiversity changes because of their enormous popularity amongst plant enthusiasts worldwide and widespread distribution (Newman et al. 2007). Unfortunately, the number of professional orchid taxonomists is dwindling. On the other hand, wildlife photography has been on the rise in the last decades because of the improved technology of cameras, lower costs and more accessible web-based portals to store and share photographs online. Free web-based portals such as Pbase, Facebook, Flicker, Pinterest, Instagram, Google+, SmugMug and other sites provide a platform to exchange photographs accessible to anyone, anywhere and anytime. Portals such as Google+, Yahoo’s Flicker and SmugMug also provide features like geotagging, enabling users to add additional data such as when and where a photograph was made in the field.

The downside of photographs uploaded by orchid enthusiasts is that plants are often incompletely or wrongly identified, especially when it concerns orchids for which no comprehensive, up-to-date taxonomic information is available. To correctly identify a plant species, plants need to be keyed out with the help of field guides, containing a description or key of all species known to occur in an area. Plant identification can be challenging, especially for novices when they have to use dichotomous keys filled with specialistic terms (Mangold 2013). With the onset of the internet era, online and real-time information can be shared, including interactive online keys for species identification. Several software packages for making interactive online keys by converting paper-printed dichotomous keys into computer-aided interactive keys are already available such as LINNAEUS 2.0, Lucid or FRIDA (Martellos 2010; Lindsay and Middleton 2009; Farr 2006). For Orchidaceae, online identification keys have already been developed for species of European orchids and the genera *Cypripedium* L. and *Vanda* Jones ex R.Br. using the Lucid3 and Xper3 platforms at the University of Basel (https://orchid.unibas.ch/index.php/en/orchidinfos/orchid-keys).The interactive keys produced with these programmes are much more user-friendly than traditional keys. They can therefore be used by a broad range of users ranging from novice plant enthusiasts up to professionals. With more accessible identification keys, it will become increasingly easy for novice users to identify plants photographed in the wild correctly.

Better identification of plants in the wild is especially needed for overlooked taxa. A prime example of such are the necklace orchids (Coelogyninae), a popular group often seen in cultivation because of their showy flowers. They belong to subfamily Epidendroideae and comprise a total of 16 genera (Gravendeel et al. 2001; Gravendeel et al. 2005; Kosina and Szkudlarek 2015). *Glomera* Blume is one of the least known genera of the necklace orchids. Species of this genus are rarely cultivated. When not in flower, most species resemble a small ericaceous shrub rather than an orchid and, regarding biomass, *Glomera* is one of the predominant orchid genera in the montane forests of New Guinea (www.orchids.naturalis.nl). A total of 169 species are known of *Glomera* after 27 species of *Glossorhyncha* Ridl. were united under *Glomera* in 2016 (Shaw 2016; Govaerts et al. 2018). The key characteristics of the genus are the elongated, often branching stem with many leaves that are enveloped by warty sheaths at the base. These species mainly occur in New Guinea but some have expanded their distribution up to Fiji, the Philippines and the New Hebrides. Most are epiphytes or terrestrials in either lowland or montane rainforest up to subalpine environments. Flowers are mostly white, but some species have orange, salmon-pink or green-coloured flowers. Inflorescences are usually single-flowered, but some species have multiple flowered inflorescences.

Traditional keys that already exist for *Glomera* are in the English and German languages only and either restricted to genus level or specific geographical regions. Examples include keys for Fiji (Kores 1989) and Papua New Guinea (van Royen 1979; www.orchids.naturalis.nl). With this publication, we present an up-to-date and accessible multilingual interactive key using the Linnaeus NG platform for identification of all species of *Glomera* in Southeast Asia.

## Software technical specification

Linnaeus NG (http://linnaeus.naturalis.nl/) is a web-based species information management system. Linnaeus NG has several modules such as species and additional features such as media (in which distribution maps and illustrations can be found) and two types of keys. For this study, a single entry key and multi-entry key were built. Linnaeus NG has been developed using open source techniques (PHP, MySQL) and is hosted in a Linux environment. On the client-side, project administrators interact with the programme through a web browser. A recent version of all major browsers is supported for regular platforms and tablets. Currently, Linnaeus NG is proprietary software; updates and changes can only be made in agreement with the Naturalis Biodiversity Center. However, access to Linnaeus NG is not limited to employees or associates of Naturalis Biodiversity Center and can be granted on request.

### Conditions of use

Linnaeus NG version 2.5 is free to use for personal and non-commercial use. Data will be guaranteed for long term sustainable hosting if they are complying with national standards in research and education. The data must be sharable and free to access and, in later stages, the developer may include adequately licensed content in Bioportal (http://bioportal.naturalis.nl/).

### User interface

Users can access the key at http://glomera.linnaeus.naturalis.nl and it can be used online using any web browser. No additional software is required. The interface was designed to be able to access from any device with flexible layout. The navigation menu is shown on the left side. The menu includes an index, species list, single-access key, multi-access key, two language options (English or Bahasa Indonesia) and a glossary. A user can directly search for a species by using the search box on the top. If the user does not yet have a clue about the identity of the species, a single-access key is available with 166 steps to help with the identification process. A multi-access key is also provided, in which remaining choices with 100% fit only are indicated at every step. A glossary is present to help novice users to understand terms used in the descriptions and keys.

### Data

**Figure 1.**
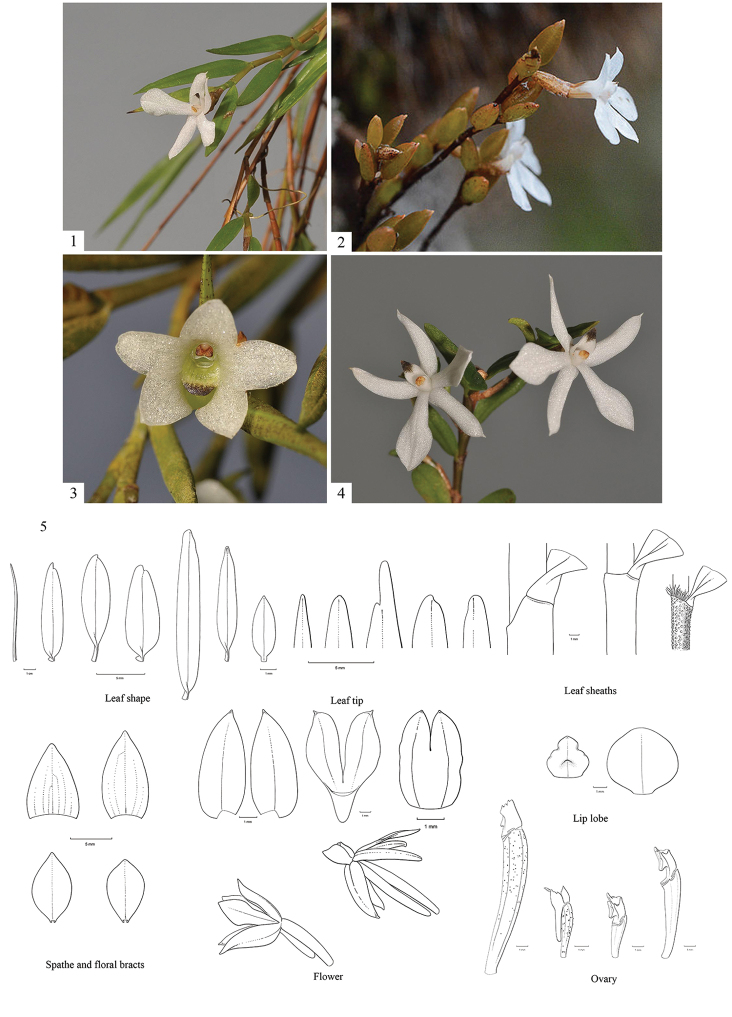
Illustrations of a selection of key characters used in the identification keys. **1***Glomeraacutiflora* (Schltr.) J.J.Sm. with green leaves (photograph by Rogier van Vugt) **2***Glomera* sp. with reddish-brown leaves (photograph by fotosynthesys deposited on FLICKR) **3***Glomerapungens* (Schltr.) J.J.Sm. with upright flowers (photograph by Rogier van Vugt) **4***Glomerahamadryas* (Schltr.) J.J.Sm. with flowers turned up-side-down (photograph by Rogier van Vugt) **5** Various shapes of the leaf blade, leaf tip, leaf sheath, leaf spathe, floral bract, entire flower, sepals, petals, lip and ovary present in *Glomera* and *Glossorhyncha* (illustrations by Esmée Winkel).

Morphological characters used in the interactive key (Figure 1; Table 2) were initially selected from already existing dichotomous keys. Not all characters turned out to be clear to both advanced and novice users, though, so the number was reduced to a final selection after tests of preliminary versions of the key by students of the annual Orchid Biology Course taught at Basel University in 2017. Students were provided with a similar set of specimens and we monitored if they were able to come up with the correction identification within half an hour and which characters were considered easy to use and which were not. Both keys were constructed independently. For the single entry key, it is possible to start at any step and, if you want to start again, you have to click on step 1. For the multi-entry key, you have to select the option ‘Start all over’ if you want to start again. All character states used are illustrated by images.

Apart from morphology, geographical distribution and ecology can be informative as well. The key therefore also includes a few of these non-morphological characters (Table 3).

**Table 1.** The 169 species of *Glomera* and their distributions included in the keys in alphabetical order.

Species	Distributions
*Glomeraacicularis* Schltr.	Papua New Guinea
*G.acuminata* J.J.Sm	Indonesia, Papua New Guinea
*G.acutiflora* (Schltr.) J.J.Sm.	Papua New Guinea
*G.adenandroides* (Schltr.) J.J.Sm.	Papua New Guinea
*G.adenocarpa* (Schltr.) J.J.Sm.	Indonesia, Papua New Guinea
*G.affinis* J.J.Sm.	Indonesia, Papua New Guinea
*G.albiviridis* P.Royen	Indonesia, Papua New Guinea
*G.altigena* (P.Royen) J.M.H.Shaw	Papua New Guinea
*G.altomontana* (Gilli) J.M.H. Shaw	Papua New Guinea
*G.amboinensis* (Ridl.) J.J.Sm.	Indonesia, Papua New Guinea, Bismarck Islands
*G.ambricaulis* (P.Royen) J.M.H. Shaw	Papua New Guinea
*G.ambuensis* (P.Royen) J.M.H. Shaw	Papua New Guinea
*G.angiensis* J.J.Sm.	Indonesia
*G.antaresensis* (P.Royen) J.M.H. Shaw	Indonesia, Papua New Guinea
*G.appendiculoides* Ormerod.	Papua New Guinea
*G.asperata* Schltr.	Papua New Guinea
*G.aurea* Schltr.	Indonesia, Papua New Guinea
*G.bambusiformis* Schltr.	Papua New Guinea
*G.bismarckiensis* J.J.Sm.	Papua New Guinea
*G.bougainvilleana* Ormerod	Papua New Guinea
*G.brachychaete* (Schltr.) J.J.Sm.	Papua New Guinea
*G.brassii* Ormerod.	Papua New Guinea
*G.brevipetala* J.J.Sm.	Indonesia, Papua New Guinea
*G.caespitosa* (P.Royen) J.M.H. Shaw	Papua New Guinea
*G.calocephala* Schltr.	Papua New Guinea
*G.carnea* J.J.Sm.	Indonesia
*G.carolinensis* L.O. Williams	Republic of Kiribati
*G.celebica* (Schltr.) J.J.Sm.	Indonesia
*G.chlorantha* (P.Royen) J.M.H.Shaw	Papua New Guinea
*G.compressa* J.J.Sm.	Papua New Guinea
*G.confusa* J.J.Sm.	Papua New Guinea
*G.conglutinata* J.J.Sm.	Indonesia
*G.crispa* (P.Royen) J.M.H.Shaw	Indonesia
*G.cristata* (P.Royen) J.M.H.Shaw	Papua New Guinea
*G.cyatheicola* P.Royen	Papua New Guinea
*G.dekockii* J.J.Sm.	Papua New Guinea
*G.dentifera* J.J.Sm.	Indonesia
*G.dependens* (Schltr.) J.J.Sm.	Papua New Guinea
*G.diffusa* (P.Royen) J.M.H.Shaw	Indonesia
*G.diosmoides* (Schltr.) J.J. Sm.	Papua New Guinea
*G.dischorensis* (Schltr.) J.J.Sm.	Papua New Guinea
*G.distichifolia* Ormerod	Vanuatu
*G.dubia* J.J.Sm.	Indonesia
*G.elegantula* (Schltr.) J.J.Sm.	Indonesia, Papua New Guinea
*G.emarginata* Kores	Fiji
*G.ericifolia* Ridl.	Indonesia
*G.erythrosma* Blume	Indonesia
*G.flaccida* (Schltr.) J.J.Sm.	Papua New Guinea
*G.flamulla* Schltr.	Papua New Guinea
*G.fluviatilis* (P.Royen) J.M.H.Shaw	Papua New Guinea
*G.fransseniana* J.J.Sm.	Indonesia
*G.fruticula* J.J.Sm.	Papua New Guinea
*G.fruticulosa* Schltr.	Papua New Guinea
*G.fusca* Schltr.	Papua New Guinea
*G.fuscosetosa* Schuit. & de Vogel	Papua New Guinea
*G.gamosepalata* P.Royen	Indonesia
*G.geelvinkensis* J.J.Sm.	Indonesia
*G.geminata* Ormerod.	Indonesia
*G.glomeroides* (Schltr.) J.J.Sm.	Papua New Guinea
*G.goliathensis* J.J.Sm.	Indonesia
*G.graminifolia* Schltr.	Papua New Guinea
*G.grandiflora* J.J.Sm.	Indonesia
*G.grandilabella* (P.Royen) J.M.H.Shaw	Papua New Guinea
*G.hamadryas* (Schltr.) J.J.Sm.	Indonesia, Papua New Guinea
*G.hubrechtiana* J.J.Sm.	Indonesia
*G.hunsteiniana* (Schltr.) J.J.Sm.	Papua New Guinea
*G.imitans* (Schltr.) J.J.Sm.	Papua New Guinea
*G.inconspicua* J.J.Sm.	Indonesia, Papua New Guinea
*G.inflata* (Schltr.) J.J.Sm.	Papua New Guinea
*G.jabiensis* J.J.Sm.	Indonesia
*G.kamay-nolomi* Ormerod.	Papua New Guinea
*G.kaniensis* Schltr.	Papua New Guinea
*G.kanke* P.Royen	Indonesia, Papua New Guinea
*G.kerewensis* (P.Royen) J.M.H.Shaw	Papua New Guinea
*G.keysseri* (Schltr.) J.M.H.Shaw	Papua New Guinea
*G.keytsiana* J.J.Sm.	Indonesia
*G.kuperensis* Ormerod.	Papua New Guinea
*G.lancipetala* J.J.Sm.	Indonesia
*G.latilinguis* J.J.Sm.	Indonesia
*G.latipetala* (Schltr.) J.J.Sm.	Papua New Guinea
*G.ledermannii* (Schltr.) J.J.Sm.	Papua New Guinea
*G.leucomela* (Schltr.) J.J.Sm.	Papua New Guinea
*G.longa* (Schltr.) J.J.Sm.	Papua New Guinea
*G.longicaulis* J.J.Sm.	Indonesia, Papua New Guinea
*G.macdonaldii* (Schltr.) J.J.Sm.	Papua New Guinea, New Caledonia, New Hebrides, Fiji
*G.macrantha* J.J.Sm.	Papua New Guinea
*G.macrophylla* Schltr.	Papua New Guinea
*G.manicata* J.J.Sm.	Papua New Guinea
*G.mayuensis* Ormerod	Papua New Guinea
*G.melanocaulon* Schltr.	Papua New Guinea
*G.merrillii* Ames	The Philippines
*G.microphylla* J.J.Sm.	Indonesia
*G.minjensis* (P.Royen) J.M.H.Shaw	Papua New Guinea
*G.minutigibba* J.J.Sm.	Indonesia
*G.montana* Rchb.f.	Papua New Guinea, Solomon, Fiji, Samoa, Vanuatu
*G.monticuprina* (P.Royen) J.M.H.Shaw	Indonesia
*G.muscicola* (P.Royen) J.M.H.Shaw	Indonesia
*G.myrtillus* (Schltr.) Schuit. & de Vogel	Papua New Guinea
*G.nana* (Schltr.) J.J.Sm.	Papua New Guinea
*G.neohibernica* Schltr.	Papua New Guinea
*G.nigricans* (P.Royen) J.M.H.Shaw	Papua New Guinea
*G.nigrilimbata* P. Royen	Papua New Guinea
*G.nigrimarginata* (P.Royen) J.M.H.Shaw	Papua New Guinea
*G.noroma* (P.Royen) J.M.H.Shaw	Papua New Guinea
*G.obovata* (Schltr.) J.J.Sm.	Papua New Guinea
*G.obtusa* Schltr.	Papua New Guinea
*G.oligantha* Schltr.	Indonesia
*G.palustris* J.J.Sm.	Indonesia, Papua New Guinea, Vanuatu, Solomon
G.palustrisvar.subintegra J.J.Sm.	Papua New Guinea
*G.papuana* Rolfe	Papua New Guinea
*G.parviflora* J.J.Sm.	Indonesia
*G.patens* Schltr.	Papua New Guinea
*G.pendulosa* J.M.H.Shaw	Papua New Guinea
*G.pensilis* (Schltr.) J.J.Sm.	Papua New Guinea
*G.pilifera* (Schltr.) J.J.Sm.	Papua New Guinea
*G.pinifolia* (P.Royen) J.M.H. Shaw	Indonesia
*G.platypetala* Schltr.	Indonesia
*G.pleiotricha* J.J.Sm.	Indonesia, Papua New Guinea
*G.plumosa* J.J.Sm.	Indonesia, Papua New Guinea
*G.polychaete* (Schltr.) J.J.Sm.	Papua New Guinea
*G.pseudomonanthos* Ormerod	Indonesia, Papua New Guinea
*G.pteropetala* (Schltr.) J.J.Sm.	Papua New Guinea
*G.pullei* J.J.Sm.	Indonesia, Papua New Guinea
*G.pumilio* J.J.Sm.	Indonesia
*G.pungens* (Schltr.) J.J.Sm.	Papua New Guinea
*G.retusa* J.J.Sm.	Indonesia
*G.retusimentum* J.J.Sm.	Indonesia
*G.rhombea* J.J.Sm.	Indonesia
*G.rigidula* J.J.Sm.	Papua New Guinea
*G.rubroviridis* J.J.Sm.	Indonesia
*G.saccharipanis* Ormerod.	Papua New Guinea
*G.saccosepala* J.J.Sm.	Papua New Guinea
*G.salicornioides* J.J.Sm.	Indonesia
*G.salmonea* J.J.Sm.	Indonesia, Papua New Guinea
*G.sandaveri* Ormerod.	Papua New Guinea
*G.scandens* J.J.Sm.	Indonesia
*G.schlechteriana* Mansf.	Papua New Guinea
*G.schultzei* Schltr.	Papua New Guinea
*G.scopulata* (P. Royen) J.M.H. Shaw	Indonesia
*G.secunda* J.J.Sm.	Indonesia
*G.sepalosiphon* Schuit. & de Vogel	Papua New Guinea
*G.similis* J.J.Sm.	Indonesia
*G.sororia* J.J.Sm.	Indonesia
*G.squamulosa* (Schltr.) J.J.Sm.	Papua New Guinea
*G.stenocentron* (Schltr.) J.J.Sm.	Indonesia, Papua New Guinea
* G. stolonifera *	Papua New Guinea
*G.subeciliata* J.J.Sm.	Indonesia
*G.sublaevis* J.J.Sm.	Indonesia
*G.subnivalis* J.M.H.Shaw	Indonesia
*G.subpetiolata* Schltr.	Papua New Guinea
*G.subracemosa* J.J.Sm.	Indonesia, Papua New Guinea
*G.subulata* (Schltr.) J.J.Sm.	Papua New Guinea
*G.subuliformis* J.J.Sm.	Indonesia
*G.tamiana* J.J.Sm.	Papua New Guinea
*G.tenuis* (Rolfe) J.J.Sm.	Papua New Guinea
*G.terrestris* J.J.Sm.	Indonesia, Papua New Guinea
*G.torricellensis* Schltr.	Papua New Guinea
*G.tortuosa* (P.Royen) J.M.H.Shaw	Papua New Guinea
*G.transitoria* J.J.Sm.	Indonesia
*G.triangularis* J.J.Sm.	Papua New Guinea
*G.tubisepala* (P.Royen) J.M.H.Shaw	Papua New Guinea
*G, umbrosa* P.Royen	Indonesia
*G.uniflora* J.J.Sm.	Indonesia
*G.verrucifera* Schltr.	Papua New Guinea
*G.verrucosissima* (Schltr.) J.J.Sm.	Papua New Guinea
*G.verruculosa* (Schltr.) J.J.Sm.	Papua New Guinea
*G.versteegii* J.J.Sm.	Indonesia, Papua New Guinea
*G.viridis* (Schltr.) J.J.Sm.	Papua New Guinea

**Table 2.** Morphological characters and their states used in the keys.

Plant part	Character	States
Rhizome	Division	Heavily branched; not or only sparsely branched
Leaf blade	Color	Green; reddish-brown
	Lamina	Fleshy; not fleshy
	Dots	With brown dots; without brown dots
	Tip	One lobe (obtuse or acute);
		two lobes (acute-acute, obtuse-obtuse or acute-obtuse)
Leaf sheath	Color	Green; black
	Tooth	With tooth; without tooth
	Notch	Notched; not notched
	Bristles	With bristles; without bristles
	Warts	With warts; without warts
Spathe	Warts	With warts; without warts
	Hairs	With hairs; without hairs
	Dots	With brown dots; without brown dots
Floral bract	Warts	With warts; without warts
	Hairs	With hairs; without hairs
	Dots	With brown dots; without brown dots
	Size	Longer than spathe; shorter than spathe
Inflorescence	Number of flowers	One; more than one
Flower	Color	White; green; pinkish-salmon; orange; red
	Orientation	Upright; up-side-down
	Spur length	Shorter than 10 mm; longer than 10 mm
	Spur tip	One-lobed; two-lobed
	Lateral sepals	Free; fused for more than two-thirds
	Lip	With glands on tip; without glands on tip
	Lip tip color	White; black; green; red; grey; pink
	Odor	Fragrant; not fragrant
	Sepal orientation	Straight; bent backward
	Column foot	Present; absent
	Ovary ribs	With ribs; without ribs
	Ovary dots	With brown dots; without brown dots
	Ovary warts	With warts; without warts

**Table 3.** Non-morphological characters and their states used in the keys.

Character group	Character	States
Ecology	Lifeform	Epiphyte; terrestrial
Flowering season	Months	January; February; March; April; May; June; July; August; September; October; November; December
Global distribution	Country	Indonesia; Papua New Guinea; Fiji; New Hebrides; Philippines
Distribution in Indonesia	Island	Papua; Java; Moluccas; Sulawesi
Occurrence over the elevational gradient	Altitude	Lowland; midland; highland

## Discussion

The interactive key presented here for *Glomera* of Southeast Asia encompasses more species and geographic areas than any existing key currently available for this genus. Next to English, it was also written in a language commonly used in Southeast Asia, Bahasa Indonesia, which enables a much wider group consisting of both novice and advanced users in the region to identify these orchids correctly. The main challenges to construct this key consisted of the fact that type descriptions were often rather vague and that many type collections were lost after the bombing of the herbarium of the Botanic Garden and Botanical Museum in Berlin in the second world war. Of the 169 species, a total of 52 types were lost. We therefore studied a lot of additional collections, all listed under Species, option Collection specimens on the website, to verify character states.

Compared with traditional dichotomous keys, interactive keys can be used much more easily by relatively novice users (Jacquemart et al. 2016). Users of this key can quickly survey images of remaining species after selection of a first set of characters such as flower colour and distribution. This key therefore enables much faster (i.e. seconds rather than minutes) identification of the best candidate from the remaining choice than working through a traditional key until the final result appears with little indication of the remaining potential outcomes during the identification process. When using a conventional dichotomous key, a user is not provided with an indication of the remaining taxa during the identification process and can only hope that the final outcome matches the candidate. Professional taxonomists often rely on extensive previous exposure to species during the identification process but novice users cannot fall back on this.

Our key will hopefully urge the users to further enrich the database and help update the distribution of species or detect possible new species. Pictures on the web placed there by enthusiasts are considered to be an essential source for the discovery of new data (Marshal 2008). Use of our key by more wildlife photographers will help to record the presence of species in geographic regions where they were previously overlooked. The idea of democratisation of taxonomy by involving the general public (hobbyists, naturalists, tourists) to this field could trigger higher interest in currently unexplored taxa. Recently, the taxonomy and biogeography of diving beetles in Bali could, for instance, be improved by using citizen scientists and social networks such as Facebook and WhatsApp (Suprayitno et al. 2017).

A first indication that the same might happen for *Glomera* orchids is illustrated by the fact that we could combine historical, literature-based data and recent photographs of plants taken by wildlife photographers of *G.aurea* Schltr., *G.macdonaldii* (Schltr.) J.J. Sm., *G.tubisepala* (P.Royen) J.M.H. Shaw and *G.glomeroides* Schltr. Pictures of flowering plants, taken in Papua of the first species, the Solomon islands of the second and Papua New Guinea of the third and the fourth and deposited on Flickr and SmugMug, came to our attention during this study. Once contacted by us, the photographers provided more detailed locality data and dates, which enabled us to update the distribution maps and also the flowering time of these species.

We also used our key to assign a name to a yet unknown *Glomera* species photographed. For example, on the photograph of *Glomera* sp. 2010-064 uploaded in the username PNG Collection of Smugmug, we could see details such as the shape of the leaf sheath, tooth and warts, colour and shape of the leaves and leaf apex and the colour of the flower (white with a green lip and red lip tip). After selecting all the characters that could be identified from the photograph and additional data such as location and altitude, we could reduce the number of species from 169 to 10. We ended up with identifying it as cf. *G.glomeroides* by comparing the photograph to a drawing made by Friedrich Richard Rudolf Schlechter in 1923 that accompanied the type description. The type of this species was lost in the herbarium of Berlin and no documented photograph has yet been published. The photographer could provide us with locality data in the Madang province of Papua New Guinea. With the aid of our interactive key, we could therefore simplify the process of identification of this unidentified species.

**Figure 2.**
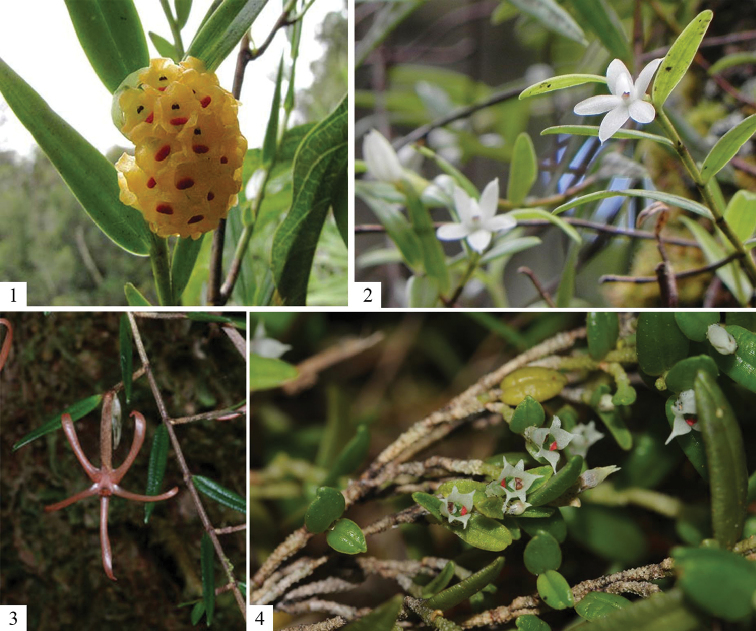
Photographs of *Glomera* species collected from online platforms. **1***Glomeraaurea* (photograph by Mehd Halaouate) **2***Glomeramacdonaldii* (photograph by Benoit Henry) **3***Glomeratubisepala* (photograph by Gary Yong Gee) **4***Glomeraglomeroides* (photograph by S.A. James).

## Conclusions

We expect that the interactive key presented here for *Glomera* of Southeast Asia will enable a higher number of people to collect more precise and more reliably identified observations of species of this overlooked orchid genus. It is user-friendly due to the many illustrations and colour photographs, encompassing a combination of historical, literature-based and recent, web-mined data of all species rather than subsets only and written in a language commonly spoken in parts of the world where these orchids occur in the wild. The key was designed for efficient use by both inexperienced and advanced orchidologists. This publication accompanies the release of version 1.0. We encourage all users to provide feedback to improve and further expand this version by contacting us by email to gain more insight into the current distribution of these overlooked orchids. This will enable us to accurately assess their conservation status. By obtaining more knowledge of the regions of distributions but without disclosing too detailed locality data, we hope to prevent extinction of these orchids in the wild.
